# Whole genome and transcriptome analyses of environmental antibiotic sensitive and multi-resistant Pseudomonas aeruginosa isolates exposed to waste water and tap water

**DOI:** 10.1111/1751-7915.12156

**Published:** 2014-09-03

**Authors:** Thomas Schwartz, Olivier Armant, Nancy Bretschneider, Alexander Hahn, Silke Kirchen, Martin Seifert, Andreas Dötsch

**Affiliations:** 1 Institute of Functional Interfaces (IFG), Campus North, Karlsruhe Institute of Technology (KIT)Hermann von Helmholtz Platz 1, Eggenstein-Leopoldshafen, D-76344, Germany; 2 Institute of Toxicology and Genetics (ITG), Campus North, Karlsruhe Institute of Technology (KIT)Hermann von Helmholtz Platz 1, Eggenstein-Leopoldshafen, D-76344, Germany; 3 Genomatix GmbHBayerstr. 85a, Munich, D-80335, Germany

## Abstract

The fitness of sensitive and resistant *Pseudomonas aeruginosa* in different aquatic environments depends on genetic capacities and transcriptional regulation. Therefore, an antibiotic-sensitive isolate PA30 and a multi-resistant isolate PA49 originating from waste waters were compared via whole genome and transcriptome Illumina sequencing after exposure to municipal waste water and tap water. A number of different genomic islands (e.g. PAGIs, PAPIs) were identified in the two environmental isolates beside the highly conserved core genome. Exposure to tap water and waste water exhibited similar transcriptional impacts on several gene clusters (antibiotic and metal resistance, genetic mobile elements, efflux pumps) in both environmental *P. aeruginosa* isolates. The MexCD-OprJ efflux pump was overexpressed in PA49 in response to waste water. The expression of resistance genes, genetic mobile elements in PA49 was independent from the water matrix. Consistently, the antibiotic sensitive strain PA30 did not show any difference in expression of the intrinsic resistance determinants and genetic mobile elements. Thus, the exposure of both isolates to polluted waste water and oligotrophic tap water resulted in similar expression profiles of mentioned genes. However, changes in environmental milieus resulted in rather unspecific transcriptional responses than selected and stimuli-specific gene regulation.

## Introduction

The increasing numbers of infections by multi-resistant bacteria turn out to be a great threat to our daily life. Bacteria develop resistance against antibiotics used in human health care, agriculture and animal husbandry or against pollutants from industry by accumulating genetic adaptations or acquisition of mobile genetic elements via horizontal gene transfer. To overcome multi-drug resistance, we have to undergo a thorough study to unravel how bacteria adapt to different habitats, to finally discover novel strategies to handle such infections.

One of the most prominent bacterial pathogens that is infamous for its high potential to develop multi-drug resistance is *Pseudomonas aeruginosa*, a Gram-negative, ubiquitous opportunistic bacterium that can cause acute and chronic infections especially in patients in intensive care or suffering from predisposing conditions like cystic fibrosis. The rate of infections in human body differs according to the site of infection as 2% on skins, 3.3% on nasal mucosa, 6.6% for the throat, 24% for fecal samples (Morrison and Wenzel, 1984). *Pseudomonas aeruginosa* is found in hospital waste water, respiratory equipment, solutions, medicines, disinfectants, sinks, mops, food mixtures and vegetables (Trautmann *et al*., 2005). An important characteristic of *P. aeruginosa* is its ability to form biofilms as an adaptation to adverse environmental conditions. The microbes attach to the surface and embed themselves in extracellular polymeric substances such as proteins (e.g. extracellular enzymes), lipids and nucleic acids (Flemming and Wingender, 2001), usually leading to increased resistance towards harsh conditions such as temperature changes, pH fluctuations, presence of antibiotics (Kwon and Lu, 2006) and immune cells of humans (Donlan and Costerton, 2002).

Sequencing of several *P. aeruginosa* strains genomes revealed that a large fraction (around 10%) of the genome is dedicated to gene regulation, which is consistent with its high versatility (Stover *et al*., 2012; Mathee *et al*., 2008). This high versatility enables evolutionary adaptations and facilitates the bacterium to colonize vigorous and diverse ecological niches. The core genome is usually highly conserved between different *Pseudomonas* strains (Mathee *et al*., 2008; Klockgether *et al*., 2011).

It has a disparate variety of metabolism; it can degrade very distinct compounds such as alcohols, fatty acids, sugars, di- and tri-carboxylic acids, aromatics, amines and amino acids, which can be used up as sources of carbon. *Pseudomonas aeruginosa* has both aerobic and anaerobic metabolism. It is capable of anaerobic metabolism by converting nitrate to nitrite (Schreiber *et al*., 2007). Additionally, the genome harbours a huge repertoire of enzymes and efflux pumps that contribute to a high intrinsic resistance towards different classes of antibiotics. Additional resistance can easily develop by mutation or horizontal gene transfer, rendering *P. aeruginosa* a common cause of multi-drug resistant infections (Breidenstein *et al*., 2011). Regular use of high amounts of antibiotics in hospitals and other practices were assumed to be the sources of origin of antibiotics in the waste water systems and responsible for supporting emergence of multi-resistant bacteria (Rizzo *et al*., 2013). These resistances may not only develop from chromosomally encoded genes but also from mobile genetic elements like plasmids or integrons (Merlin *et al*., 2011). Not only waste water systems contribute to the development of resistance in bacteria, but also pollutants from industries and agricultural activities where the antibiotics and pollutants are directly released into the environmental water like rivers and lakes, creating selective pressure on these bacteria and making them evolve as resistance strains. The sensitive strains accept the resistant genes from these resistant donors and propagate as resistant strains. The concentrations of antibiotics in waste water might not be high enough to stimulate inhibitory effects but stimulate stress response mechanisms, which contribute to horizontal gene transfer and relevant transcriptional activities. It has also been proven by mutant investigations that sub-inhibitory concentrations of antibiotics can drive the evolution of antimicrobial resistance (Pedró *et al*., 1996). It all depends on the substance, concentration and strain present in the waste water systems. There is no final suggestion about long terms effects of sub-inhibitory concentration antibiotics and other micro-pollutants. It is commonly accepted that beside the linkage between antibiotics and antibiotic resistance, co-selection and the presence of heavy metal ions in the environments contributes to increasing resistance mechanisms due to the localization of resistance genes in close neighborhood on genetic mobile elements (Seiler and Berendonk, 2012).

Beside antibiotic and heavy metal stress, starvation is another widespread adverse stimulus present in many aquatic environments where *P. aeruginosa* is found in nature (Bernier *et al*., 2013). Tap water represents an oligotrophic matrix with very low organic matter, and *P. aeruginosa* has recently been shown to persist and proliferate as biofilms in municipal drinking water distribution systems (Wang *et al*., 2012). The molecular responses of *P. aeruginosa* strains to starvation stress in tap water are so far unknown. In this study, we compared the transcriptional response of an antibiotic sensitive and a multi-resistant *P. aeruginosa* waste water isolate cultivated in municipal waste water and tap water focusing on regulatory mechanisms that could promote the development of antibiotic resistance.

## Results and discussion

Bacteria have developed highly orchestrated processes to respond to environmental stresses, which when elicited alter the cellular physiology in a manner that enhances the organism's survival and its ability to cause disease. This study focused on the behaviour of two natural isolates of *P. aeruginosa* as a Gram-negative bacterium exposed to municipal waste water containing complex mixtures of xenobiotics and, as a second scenario, exposed to tap water simulating nutrient limitation (starvation). Since bacteria have to deal with unfavourable growth conditions in addition to diverse stresses in nature, bacteria that reached the stationary growth phase were used to imitate this environment and then exposed to stress. During transition from exponential growth to stationary phase, growth becomes unbalanced especially in laboratory systems, i.e. the synthesis of different macromolecules and cell constituents do not slow down synchronically (Nyström, 2004). Thus, stationary phase is an operational definition and does not describe a specific and fixed physiological state or response of the bacteria. It is more or less a change in physiology due to, e.g. phosphate limitation or accumulation of toxic waste products. Beside the changes in morphologies of bacteria, the gene expression pattern could be altered in stationary phase. In consequence, transcriptome analyses were run with ribonucleic acid (RNA) extracted from early stationary growth phase. In the present study, two different *P. aeruginosa* isolates were exposed to water matrices containing quite different compositions. Waste water from the influent of a municipal waste water treatment plants (WWTP) is composed of complex mixtures of xenobiotics like antibiotics, other pharmaceuticals, biocide etc., whereas tap water, in opposite, contains very low level of organic matter (including xenobiotics) as a result of the intensive drinking water conditioning processes at waterworks. We analysed the transcriptional responses from two *P. aeruginosa* isolates: the antibiotic sensitive strain PA30 and the multi-resistant strain PA49. Both *P. aeruginosa* strains did neither show any differences in growth in diluted brain heart infusion (BHI) or BM2 broth nor in yields of extracted total RNA after exposure in tap water or waste water.

### Genome analyses

Large fractions of the *P. aeruginosa* genome belong to the highly conserved core genome containing only few highly variable genes (Dötsch *et al*., 2010), while most of the genetic variation between species is restricted to the so-called *accessory genome* organized in various *regions of genomic plasticity* (RGPs) (Mathee *et al*., 2008). Most of these RGPs represent mobile elements originating from horizontal gene transfer and include transposons, phages, plasmids and genomic islands, which are a major source of resistance genes (Battle *et al*., 2009; Kung *et al*., 2010; Klockgether *et al*., 2011). The large amount of homology between the core regions of different *P. aeruginosa* strains enabled us to employ the genomic sequences of strain PAO1 chromosome and a selection of genomic islands as a blueprint for *de novo* assembly. The resulting draft genomes consist of 207 contigs with a total length of 6.77 Mb for the strain PA30 and 269 contigs with 7.01 Mb for strain PA49 respectively (Table S1).

**Figure 1:**
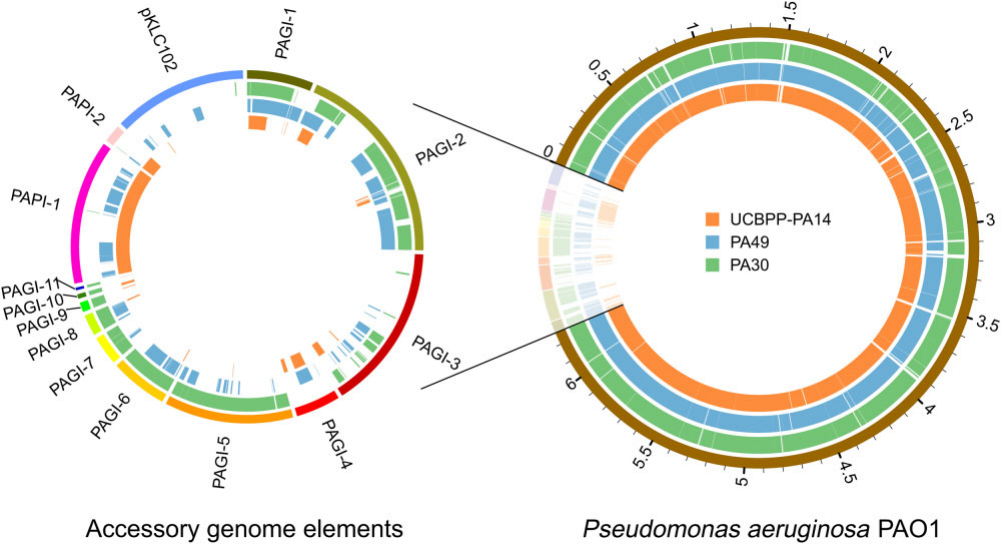
Coverage of genomic reference sequences by the newly assembled genomes. The reference sequences that were used for the hybrid *de novo* assembly are displayed on the outer circles (diagram to the right) with an additional display of the accessory elements alone (missing the PAO1 chromosome, diagram to the left). Regions that are covered by the contigs of strains PA30 and PA49 or overlap with the chromosome of another reference strain PA14 are highlighted by coloured areas in the concentric inner circles as specified by the color legend.

An alignment of the contigs with *P. aeruginosa* reference strain PAO1 showed a huge overlap of 95.8% for PA30 and 96.4% for PA49 (Fig. 1; Table S2), reflecting the highly conserved character of the *P. aeruginosa* core genome. Comparing the contigs with the genome islands that were used in the alignment process revealed a distinct pattern of accessory genomic elements for the two strains covering large fractions of the various genomic islands (Fig. 1; Table S2). Strain PA30 contains full length or near-full length sequences of PAGI-5 to PAGI-11, larger fractions of PAGI-1 and PAGI-2 and several regions of PAGI-3, whereas only insignificant fractions of the remaining genomic elements occurred. In case of the multi-resistant strain PA49, all genomic islands except the smaller PAGI-9 to PAGI-11 were covered at varying percentages (Table S2). The scattered distribution of regions within the genomic islands that actually showed homology with PA49 contigs may be partially explained by incomplete sequence assembly. However, the fact that both the contigs and the genomic island reference sequences contained a large amount of non-overlapping regions (data not shown) suggests that at least in some cases, the accessory elements found in PA30 and PA49 only partially contain sequences that are homologous to the genomic islands and also include a substantial amount of new and previously uncharacterized sequences.

**Figure 2:**
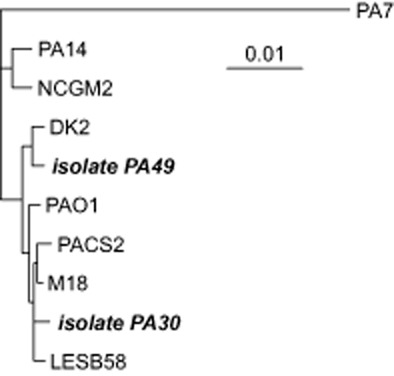
Multi-locus sequence typing phylogenetic tree. Phylogenetic relations of the two newly sequenced strains PA30 and PA49 (bold) with eight previously published genomes of *P. aeruginosa*. The phylogenetic tree is based on seven genes that are commonly used for MLST scheme by Curran and colleagues (2004).

Since the genomes of PA30 and PA49 were nearly completely covered, the sequence types according to the multi-locus sequence typing (MLST) scheme by Curran and colleagues (2004) could be determined, enabling a phylogenetic classification of the two strains. As demonstrated by the phylogenetic tree (Fig. 2), PA30 and PA49 are members of the lineage that includes the type strain PAO1 and some recently sequenced strains. The question about their origin is open, since the sampling sites were influenced by hospital and housing waste waters. Selective pressures like the presence of antibiotics and other environmental criteria are a general concept that refers to many factors that create an evolutionary landscape and allow organisms with novel mutations or newly acquired characteristics to survive and proliferate (Kümmerer, 2009). There is evidence that even in sub-inhibitory concentration, antibiotics or other xenobiotics may still exert their impact on microbial communities (Goh *et al*., 2002; Davies *et al*., 2006). The direct link between antibiotics or heavy metal ions and development/selection of resistance mechanisms is obvious and manifold described (Seiler and Berendonk, 2012; Rizzo *et al*., 2013). The impact of other harsh environmental conditions on resistance activities, recombination and horizontal gene transfer remains to be determined. Long-term effects of environmental exposure to low levels of antibiotics like these present in surface waters or in the outflow of sewage plants are also still unknown.

**Figure 3:**
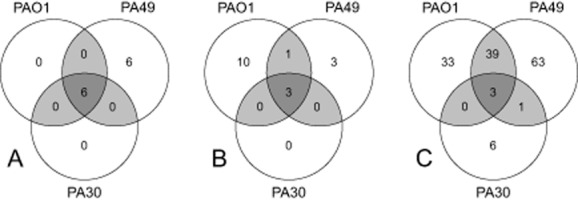
Comparison of specific gene classes found in the genomes of PA30 and PA49 with type strain PAO1. The circles of this Venn Diagram contain the numbers of genes that were predicted from the genome sequence of the two newly sequenced strains, in comparison with the known genes of the PAO1 reference genome. PAO1 genome annotation was taken from www.pseudomonas.com (Winsor *et al*., 2011).A. Genes involved in antibiotic resistance (excluding efflux pumps).B. Genes involved in metal ion resistance.C. Gene involved in genetic mobility – transposases, integrases, recombinases and conjugation-related proteins.

The prediction of protein coding sequences (CDS) yielded for both strains a comparatively large number of genes, about 99% of which were successfully annotated according to their best-hit BLAST alignments (Table S1). The vast majority of the predicted genes were found in both strains and also in the PAO1 reference genome (5262 genes), representing the conserved core genome of *P. aeruginosa*. Regarding the development of multi-drug resistance, *P. aeruginosa* is known for its high intrinsic resistance that is caused by a combination of low membrane permeability, efflux pumps and resistance genes encoded in the core genome (Nikaido, 2001; Schweizer, 2003), together with the potential to develop high-level resistance by accumulation of small mutations (Fajardo *et al*., 2008; Dötsch *et al*., 2009; Martinez *et al*., 2009; Alvarez-Ortega *et al*., 2010; Breidenstein *et al*., 2011; Bruchmann *et al*., 2013). However, the most obvious cause of multi-drug resistance is the acquisition of resistance genes by horizontal gene transfer (Davies and Davies, 2010). Therefore, we performed a blast search of the predicted genes of the two strains in the Comprehensive Antibiotic Resistance Database (CARD) (McArthur *et al*., 2013) and scanned both genomes for genetic variations of intrinsic resistance determinants. In a previous work, strain PA49 was found to be resistant towards the antibiotics gentamicin (GM), amikacin (AN), azlocillin (AZ), ceftazidime (CAZ), piperacillin/tazobactam (PT), ciprofloxacin (CIP) and imipenem (IPM) (Schwartz *et al*., 2006). Searching its genome for resistance determinants revealed the presence of one aminoglycoside acetyltransferase of the AAC(6’)-type, two aminoglycoside adenylyltransferases of type ANT(2'’) and ANT(3'’) and one VIM metallo-beta-lactamase (Table 1). Two additional genes were annotated as beta-lactamases in PA49 only by the BLAST search in the National Center for Biotechnology Information (NCBI) non-redundant (nr) protein database but not found in the CARD database (Fig. 3A). Taken together, these genes confer resistance towards a wide range of aminoglycosides and beta-lactam antibiotics, explaining the resistance towards GM, AN, AZ, CAZ and PT. Fluoroquinolones like CIP target the DNA gyrase and Topoisomerase IV enzyme complexes, and high-level resistance towards these antibiotics is often caused by sequence variations of the two subunits GyrA (gyrase) and ParC (topoisomerase) (Ruiz, 2003) and indeed, both proteins contained a single amino acid exchange in the resistance determining region (Table 1). These two mutations represent the most common type of variations found in fluoroquinolone resistant isolates of *P. aeruginosa* and have recently been shown to be sufficient for the development of high-level resistance towards CIP (Bruchmann *et al*., 2013). Finally, a frameshift mutation in the outer membrane porin OprD was found that is likely to cause misfolding or decreased functionality of the protein. Defective mutations of OprD are known to cause resistance towards carbapenems including IPM in combination with intrinsic beta-lactamases and efflux pumps (Pirnay *et al*., 2002). Of note, the strain PA30 that is sensitive towards all these antibiotics did not contain any known horizontally acquired resistance genes and harboured wild-type alleles of the target genes *gyrA*, *parC* and *oprD* (Table 1). In summary, these results provide a comprehensive explanation for the resistance phenotype covering all the antibiotics that were tested, since all resistance determining genes and alleles (besides the ones intrinsic to *P. aeruginosa*) were exclusively found in PA49 (Fig. 3A; Table 2).

**Table 1:** Comparison of antibiotic resistance determinants found in the genomes of PA30 and PA49. Identifiers state PAO1 gene IDs or RefSeq Accession where applicable. Genotypes refer to presence or absence or genes or specific alleles with ‘wt’ indicating the genotype found in the reference strains PAO1

Gene ID/accession	Gene name	Resistance type	PA30 genotype	PA49 genotype	Affected antibiotics^a^
gi|32470063	aac(6’)-Ib	AAC(6’)	–	Present	Aminoglycosides (GM, AN)
gi|378773997	aadB	ANT(2'’)	–	Present	Aminoglycosides (GM, AN)
gi|489251134	blaVIM-2	VIM	–	Present	Beta-lactams (AZ, CAZ, PT)
gi|88853419	aadA10	ANT(3'’)	–	Present	Aminoglycosides
gi|489211498	–	AmpC	–	Present	Beta-lactams (AZ, CAZ, PT)
gi|489217979	–	Metallo-beta-lactamase	–	Present	Beta-lactams (AZ, CAZ, PT)
gi|407937916	–	Metallo-beta-lactamase	Present	–	Beta-lactams (AZ, CAZ, PT)
PA3168	gyrA	Target modification	wt	T83I	Fluoroquinolones (CIP)
PA4964	parC	Target modification	wt	S87L	Fluoroquinolones (CIP)
PA0958	oprD	Decreased permeability	Multiple SNPs	Multiple SNPs, frameshift	Carbapenems (IPM)
				insertion CC at position 279	

a Abbreviations indicate specific antibiotics. AN, amikacin; PT, Piperacillin + Tazobactam.

AZ, azlocillin; CAZ, ceftazidime; CIP, ciprofloxacin; GM, gentamicin; IPM, imipenem.

**Table 2:** Expression of genes related to antibiotic resistance (excluding efflux pumps)

		PA30		PA49	
Gene ID/accession	Product	T	W	T	W
gi|489182848	Acriflavin resistance protein	n.p.^a^	n.p.	524	416
gi|496684660	Bleomycin resistance protein	n.p.	n.p.	1808	1737
gi|489211498	Beta-lactamase	n.p.	n.p.	1028	435
gi|489251134	VIM-1 protein	n.p.	n.p.	35 957	39 477
gi|32470063	Hypothetical protein	n.p.	n.p.	50 184	48 460
gi|88853419	Aminoglycoside-modifying enzyme	n.p.	n.p.	4358	3280
gi|378773997	2.-aminoglycoside nucleotidyltransferase	n.p.	n.p.	7275	5761
gi|496684693	Glyoxalase/bleomycin resistance protein/dioxygenase	n.p.	n.p.	362	1547
gi|160901172	Acriflavine resistance protein B	n.p.	n.p.	468	408
gi|489217979	Beta-lactamase	n.p.	n.p.	109	64
PA0706	Chloramphenicol acetyltransferase	399	304	48	73
gi|489208693	Fusaric acid resistance protein	25	18	30	18
gi|116049746	Beta-lactamase	107	64	272	131
PA1129	Probable fosfomycin resistance protein	82	54	33	11
PA5514	Probable beta-lactamase	258	285	191	422
PA1959	Bacitracin resistance protein	385	925	110	219
PA4110	Beta-lactamase precursor AmpC	694	382	101	74
PA4119	Aminoglycoside 3'-phosphotransferase type IIb	113	177	37	40
PA5159	Multi-drug resistance protein	79	66	65	120
PA1858	Streptomycin 3'’-phosphotransferase	145	131	31	52
gi|407937916	beta-lactamase domain-containing protein	233	201	n.p.	n.p.

a n.p. – gene sequence is not present in this strain.

Identifiers state PAO1 gene IDs or RefSeq accession where applicable. Transcriptional activity in tap water (T) and waste water (W) was normalized for the two strains independently and is shown as normalized pseudocounts.

Both PA30 and PA49 harbour a set of genes involved in metal ion resistance that are not found in the *P. aeruginosa* core genome. Strain PA30 contains several genes encoding resistance genes related to copper, mercury and arsenic/arsenate, while most of the genes could not be found in PA49 (Fig. 3B; Table 3).

**Table 3:** Expression of genes related to metal tolerance

		PA30		PA49	
Gene ID/accession	Product	T	W	T	W
gi|489181230	Mercuric reductase	n.p.^a^	n.p.	695	892
gi|134047226	MerP	n.p.	n.p.	190	376
gi|410691713	Hg(II)-responsive transcriptional regulator MerR	n.p.	n.p.	626	2339
gi|498493737	Mercuric reductase	515	520	n.p.	n.p.
gi|134047116	MerT	65	53	132	339
gi|152989484	Mercuric resistance operon regulatory protein	239	310	n.p.	n.p.
PA2065	Copper resistance protein A precursor	253	95	654	20
PA2064	Copper resistance protein B precursor	114	59	238	12
PA0950	Probable arsenate reductase	472	615	268	451
gi|498490899	Arsenical resistance protein ArsH	102	139	n.p.	n.p.
gi|493249841	Arsenate reductase	75	97	n.p.	n.p.
gi|493264869	Arsenic transporter	119	196	n.p.	n.p.
gi|493249844	ArsR family transcriptional regulator	486	235	n.p.	n.p.
gi|495544062	Copper oxidase	1420	203	n.p.	n.p.
gi|493265806	Copper resistance protein B	612	100	n.p.	n.p.
gi|386717894	Copper resistance protein C	358	141	n.p.	n.p.
gi|489188126	Copper resistance protein CopD	717	392	n.p.	n.p.

a n.p. – gene sequence is not present in this strain.

Identifiers state PAO1 gene IDs or RefSeq accession where applicable. Transcriptional activity in tap water (T) and waste water (W) was normalized for the two strains independently and is shown as normalized pseudocounts.

The extent of the accessory genomes found in PA30 and PA49 point towards a high incidence of horizontal gene transfer in the evolutionary history of these strains. Therefore, we also searched the annotated genomes for genes associated with genomic mobility, mostly classified as recombinases, transposases, integrases or conjugative elements. Since mobile genetic elements *per definition* belong to the accessory genome, it is not surprising that nearly all genes associated with genetic mobility that were found in PA30 and PA49 are not present in the genome of PAO1 (Fig. 3C). Both strains contain a large number of mobility genes (75 in PA30, 103 in PA49) (Table 4).

**Table 4:** Expression of genes related to genetic mobility and horizontal gene transfer

		PA30		PA49	
Gene ID/accession	Product	T	W	T	W
gi|386063806	Conjugal transfer protein TrbJ	n.p.^a^	n.p.	4	7
gi|446855879	Conjugal transfer protein TrbE	n.p.	n.p.	58	33
gi|446855879	Conjugal transfer protein TrbE	n.p.	n.p.	26	21
gi|386063808	Conjugal transfer protein TrbC	n.p.	n.p.	10	16
gi|51492563	TnpA	n.p.	n.p.	481	609
gi|66045904	TnpR resolvase	n.p.	n.p.	2370	3179
gi|19352419	Putative transposase	n.p.	n.p.	3570	2090
gi|498491672	Excisionase family DNA binding domain-containing protein	n.p.	n.p.	263	432
gi|496684679	Conjugal transfer coupling protein TraG	n.p.	n.p.	22	8
gi|190573650	Transposase	n.p.	n.p.	218	206
gi|190572552	Transposase IS3	n.p.	n.p.	189	156
gi|497209012	Transposase	n.p.	n.p.	0	0
gi|495918616	Integrase	n.p.	n.p.	187	126
gi|37958842	Putative transposase	n.p.	n.p.	202	452
gi|386063806	Conjugal transfer protein TrbJ	n.p.	n.p.	29	5
gi|446923490	Conjugal transfer protein TrbL	n.p.	n.p.	66	40
gi|410690825	Transposase	n.p.	n.p.	837	2156
gi|319765135	Transposase TniA	n.p.	n.p.	874	1657
gi|491446119	TniQ	n.p.	n.p.	448	546
gi|490385992	Transposase	n.p.	n.p.	26	30
gi|497303964	Integrating conjugative element protein	n.p.	n.p.	4	6
gi|497081867	Conjugative transfer region protein	n.p.	n.p.	2	2
gi|497303936	Integrating conjugative element protein	n.p.	n.p.	0	0
gi|497081861	Integrating conjugative element protein	n.p.	n.p.	13	11
gi|493535069	Integrating conjugative element protein. PFL_4709 family	n.p.	n.p.	19	19
gi|497081686	Integrase family protein	n.p.	n.p.	223	259
gi|133756449	TniA	n.p.	n.p.	498	866
gi|289064112	TniQ transposition protein	n.p.	n.p.	57	45
gi|410609201	Transposase	n.p.	n.p.	2789	2351
gi|446985433	Integrase	n.p.	n.p.	0	1
PA4797	Probable transposase	n.p.	n.p.	1294	879
gi|496684684	Conjugal transfer protein TrbE	n.p.	n.p.	28	27
gi|496684684	Conjugal transfer protein TrbE	n.p.	n.p.	13	15
gi|496684684	Conjugal transfer protein TrbE	n.p.	n.p.	7	12
gi|496684685	Conjugal transfer protein TrbJ	n.p.	n.p.	1	0
gi|496684685	Conjugal transfer protein TrbJ	n.p.	n.p.	14	14
gi|330503729	Transposase IS4	n.p.	n.p.	91	80
gi|496684687	Conjugal transfer protein TrbL	n.p.	n.p.	79	43
gi|256367798	Transposase	n.p.	n.p.	15674	13666
gi|496684681	Conjugal transfer protein TrbB	n.p.	n.p.	1	1
gi|116050177	Transposase Tn4652	n.p.	n.p.	596	487
gi|116050169	Recombinase	n.p.	n.p.	553	1182
gi|152984407	TnpT protein	n.p.	n.p.	180	266
gi|12698413	Transposase B	n.p.	n.p.	50	71
gi|498341535	Integrase [Pseudomonas fragi]	n.p.	n.p.	453	462
gi|489250021	Conjugal transfer protein TrbL	n.p.	n.p.	1	0
gi|496684681	Conjugal transfer protein TrbB	n.p.	n.p.	1	2
gi|496684682	Conjugal transfer protein TrbC	n.p.	n.p.	0	2
gi|497207593	Integrase	n.p.	n.p.	3508	2908
gi|489182829	Conjugative transfer protein TrbI	n.p.	n.p.	87	59
gi|386063802	Conjugative transfer protein TrbG	n.p.	n.p.	25	15
gi|386063802	Conjugative transfer protein TrbG	n.p.	n.p.	29	14
gi|386063803	Conjugal transfer protein TrbF	n.p.	n.p.	22	3
gi|386063804	Conjugal transfer protein TrbL	n.p.	n.p.	4	0
gi|505461140	Shufflon-specific recombinase	n.p.	n.p.	339	328
gi|497074269	Integrating conjugative element protein pill. pfgi-1	n.p.	n.p.	7	5
gi|485834156	Transposase	n.p.	n.p.	632	428
gi|446195994	Transposase	n.p.	n.p.	374	259
gi|496684690	Conjugal transfer protein TrbI	n.p.	n.p.	14	9
gi|496684690	Conjugal transfer protein TrbI	n.p.	n.p.	15	23
gi|496684689	Conjugal transfer protein TrbG	n.p.	n.p.	8	14
gi|495242526	Conjugal transfer protein TrbF	n.p.	n.p.	11	4
gi|17547297	Conjugal transfer protein TrbL	n.p.	n.p.	1	0
gi|3688518	Putative transposase	n.p.	n.p.	968	1026
gi|491446843	Integrase	n.p.	n.p.	48	67
gi|152987984	Conjugal transfer protein TrbD	n.p.	n.p.	2	5
gi|493518183	Conjugal transfer protein TrbC	n.p.	n.p.	3	1
gi|489201924	Conjugal transfer protein TrbB	n.p.	n.p.	3	1
gi|330824177	Conjugal transfer protein TrbB	n.p.	n.p.	8	2
gi|489194682	Conjugal transfer protein TraG	n.p.	n.p.	6	8
gi|92112121	TnpA transposase	n.p.	n.p.	2857	1238
gi|505462488	Transposase mutator family protein	n.p.	n.p.	483	234
gi|472324076	Site-specific recombinase XerC	n.p.	n.p.	187	201
gi|512557653	Transposase	55	51	57	30
gi|489232756	Transposase component	7	8	13	5
gi|495332841	Transposase	159	157	n.p.	n.p.
gi|410693866	Transposase of ISThsp18. IS1182 family	2730	2154	n.p.	n.p.
gi|392420288	Integrating conjugative element ParB	28	75	4	11
gi|330824345	Integrating conjugative element protein	83	168	11	25
gi|330824346	Integrase	31	67	36	24
gi|512557590	Integrating conjugative element protein PilL. PFGI-1 class	8	6	n.p.	n.p.
gi|490375364	Integrating conjugative element protein	15	35	n.p.	n.p.
gi|339493279	Conjugal transfer protein TraG	109	96	n.p.	n.p.
gi|498491306	Integrating conjugative element protein	6	2	9	9
gi|497303938	Integrating conjugative element membrane protein	3	8	7	2
gi|392420336	Conjugal transfer protein	8	9	n.p.	n.p.
gi|330824396	Integrating conjugative element protein	26	22	n.p.	n.p.
gi|330824398	Integrating conjugative element protein	13	18	n.p.	n.p.
gi|497303929	Conjugative transfer ATPase	73	44	32	19
gi|498491292	Integrating conjugative element protein	39	22	4	16
gi|410471275	Phage-related integrase	442	603	n.p.	n.p.
PA3738	Integrase/recombinase XerD	177	231	122	275
gi|489224124	Integrase	1148	1068	n.p.	n.p.
gi|489224128	Integrase	1072	1106	n.p.	n.p.
gi|498248343	Transposase	124	98	n.p.	n.p.
gi|496762510	Conjugal transfer protein TrbJ	359	253	n.p.	n.p.
gi|489229539	Conjugal transfer protein TrbL	417	610	n.p.	n.p.
gi|489229536	Conjugal transfer protein TrbJ	479	538	n.p.	n.p.
gi|489229532	Integrase	634	593	n.p.	n.p.
gi|157420224	TnpA	0	0	1224	1485
gi|489225709	Integrase	230	160	137	63
gi|353334486	IncI1 plasmid conjugative transfer ATPase PilQ	241	176	84	59
gi|148807320	Site-specific recombinase	348	392	n.p.	n.p.
gi|152985999	Conjugal transfer protein TraG	565	416	207	167
gi|490477548	Conjugal transfer protein	98	44	19	16
gi|498491413	Conjugative transfer ATPase	510	328	218	122
gi|386064726	Tyrosine recombinase XerC	431	444	237	196
gi|386056644	Integrase	71	85	44	61
gi|446985433	Integrase	109	80	172	178
gi|163856484	Transposase	115	68	n.p.	n.p.
gi|496684672	Conjugal transfer protein TraF peptidase	9	2	n.p.	n.p.
gi|187940137	Phage integrase family protein	187	135	n.p.	n.p.
gi|121595234	Conjugal transfer coupling protein TraG	36	26	97	110
gi|386063809	Conjugal transfer protein TrbB	14	12	12	21
gi|492686504	Conjugal transfer protein Trbc	5	4	n.p.	n.p.
gi|387129929	Conjugal transfer protein TrbD	2	2	6	3
gi|497202778	Conjugal transfer protein TrbE	55	58	47	31
gi|489201921	Conjugal transfer protein TrbJ	47	28	11	7
gi|491446728	Conjugal transfer protein TrbL	869	474	16	8
gi|152984687	Transposase	709	388	n.p.	n.p.
gi|489221152	Integrase	645	569	n.p.	n.p.
gi|489184774	Integrase	352	296	n.p.	n.p.
gi|512563762	P-type conjugative transfer protein TrbL	15	3	n.p.	n.p.
gi|94310290	conjugal transfer protein TrbF	17	22	23	4
gi|512587667	P-type conjugative transfer protein TrbG	31	31	20	14
gi|489200991	Conjugative transfer protein TrbI	76	67	38	55
gi|490275574	IS5 Transposase. Partial	796	610	n.p.	n.p.
gi|218891103	Integrase	362	278	111	97
gi|392983596	Integrase	3054	1923	476	858
gi|489349534	Transposase IS3	327	133	n.p.	n.p.
gi|187939496	Transposase	453	382	560	641
PA1534	Recombination protein RecR	390	500	231	278
PA5280	Site-specific recombinase Sss	320	320	112	113
gi|148807435	Phage integrase	115	87	n.p.	n.p.
gi|148807434	Phage integrase	371	303	n.p.	n.p.
gi|148807431	Site-specific recombinase	598	366	488	488
gi|489214965	Integrase	999	937	226	488
gi|148807465	Phage integrase	121	139	n.p.	n.p.
gi|392420677	Transposase IS5	420	266	n.p.	n.p.
gi|94311234	Integrase	83	88	12	4
gi|493264878	Conjugal transfer protein TraG	81	100	101	58
gi|489180720	Conjugal transfer protein	6	1	2	2
gi|386717922	Conjugal transfer protein	76	77	54	53
gi|489215831	Integrase	347	368	1107	3025
gi|498490909	Integrating conjugative element relaxase. PFGI-1 class	212	126	181	129
gi|493264934	Transposase IS204	332	292	n.p.	n.p.
gi|493265821	Transposase	303	257	n.p.	n.p.
gi|489180871	Integrase	521	582	181	240

a n.p. – gene sequence is not present in this strain.

Identifiers state PAO1 gene IDs or RefSeq accession where applicable. Transcriptional activity in tap water (T) and waste water (W) was normalized for the two strains independently and is shown as normalized pseudocounts.

### Transcriptome analyses

In order to investigate the impact of waste water and tap water on the transcriptional activities, we performed RNA sequencing on both the sensitive and multi-resistant *P. aeruginosa* strain. The *de novo* assembled genomes were used as references for the mapping of reads obtained from RNA sequencing. In total, 95% of the reads mapped to the genome reference, which is comparable to results for RNA sequencing of known genomes and indicates a high quality and completeness of the two assembled genomes (Table S3). Between 1.5 and 3.6 million reads mapped uniquely to coding regions, yielding a median read count per gene of 54 to 130 and was sufficient for an in depth analysis.

Both strains, PA30 and PA49, were exposed to tap water and waste water, and differential gene expression was analysed between the different water matrices as well as between the two strains. Upon exposure to waste water, 222 genes were at least fourfold differentially expressed in strain PA30 (94 upregulated, 128 downregulated) as compared with tap water exposure (Table S4). Most of the differentially expressed genes encode for hypothetical proteins. Investigating whether any functional groups of genes were significantly over-represented among the differentially expressed genes, we performed an enrichment analysis of gene ontology (GO) terms. Genes that were associated with ‘copper ion binding’ (GO:0005507) and ‘potassium-transporting ATPase activity’ (GO:0008556) were significantly over-represented with six (out of 17) and three (out of three) genes being differentially expressed respectively. In strain PA49, 144 (51 upregulated, 93 downregulated, Table S5) gene showed differential expression upon exposure to the different water matrices, but no significant enrichment of GO terms was observed. A comparison of the expression of orthologous genes between PA30 and PA49 revealed a differential in the expression of 32 genes in tap water (e.g. some phenazine biosynthesis genes and a potassium-transporting ATPase, kdpABC, were upregulated in PA30), while only five gene coding for hypothetical proteins were found to be differentially expressed in waste water. This low number of differentially expressed genes between the two strains indicates a high similarity in their response to these specific environments.

**Figure 4:**
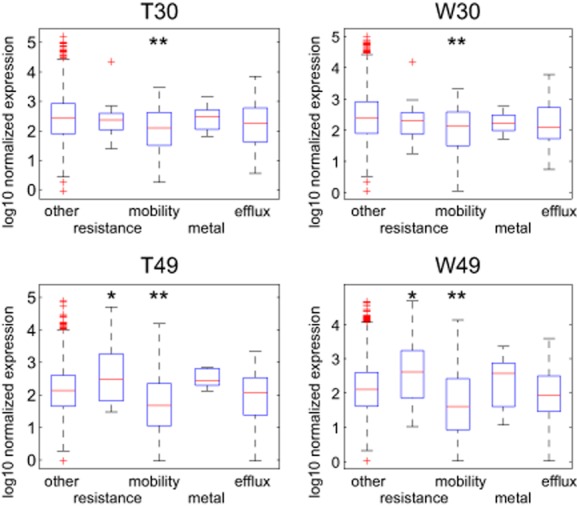
Expression of specific genes in strain PA30 and PA49 in different water matrices. Absolute gene expression values are depicted as box plots for the different samples of strain PA30 and PA49 cultivated in tap water (T) and waste water (W). Genes were manually selected by their functional classification as *resistance* (associated with modification and deactivation of antibiotics), *mobility* (associated with horizontal gene transfer and recombination), *metal* (associated with heavy metal tolerance), *efflux* (associated with multi-drug efflux pumps) or *other* (not included in any other class). Asterisks indicate a significant difference in the medians of the particular gene class and the *other* genes determined with the Mann–Whitney–Wilcoxon test (*P < 0.05; **P < 0.001).

The four horizontally acquired antibiotic resistance genes found in strain PA49 (Table 1) were transcriptionally active independent from the water matrix and therefore most likely are a main cause of the observed resistance towards a wide spectrum of aminoglycoside and beta-lactam antibiotics (Table 2). Genes associated with antibiotic resistance (not including multi-drug efflux pumps) showed a higher average expression as compared with the rest of the genome in PA49 (Fig. 4), which is obviously a result of the generally high expression of horizontally acquired resistance genes (Table 2). This tendency was independent from the water matrix and not found in the transcriptome of PA30 (Fig. 4), which lacks such additional resistance genes (Fig. 2). A common cause of antibiotic resistance in *P. aeruginosa* is the overexpression of multi-drug efflux pumps (usually termed ‘Mex’ pumps). Indeed, the genes encoding the MexCD-OprJ efflux pump were overexpressed in PA49 in response to waste water (Table 5 and Table S5). This pump system can confer resistance towards a broad spectrum of antibiotics (Poole *et al*., 1996) and thus may further contribute to the multi-resistance phenotype of PA49. The induced expression of this efflux pump specifically in waste water is indicating a specific stimulation presumably by one or multiple of antibiotics found in the used waste water or via so far unknown waste water components. However, since the expression of specific resistance genes and presence of resistance-related target mutations already sufficiently explains the broad resistance phenotype in PA49 (Table 1), the exact contribution of a MexCD-OprJ overexpression remains unclear. It should be again pointed out that the expression of resistance genes (with the exception of MexCD-OprJ) in PA49 was independent from the water matrix. Similarly, the antibiotic sensitive strain PA30 does not show any difference in expression of the intrinsic resistance determinants. Thus, the exposure of both strains to polluted waste water and oligotrophic tap water resulted in similar expression profiles of resistance genes. It seems to be obvious that changes in environmental milieus result in rather unspecific transcriptional responses than selected and stimuli-specific gene regulation.

**Table 5:** Expression of genes encoding (putative) multi-drug efflux pumps (MEX pumps)

		PA30			PA49
Gene ID/accession	Product	T	W	T	W
gi|15596434	Multi-drug resistance efflux pump	n.p.^a^	n.p.	8	6
gi|491814602	Macrolide efflux protein	n.p.	n.p.	69	85
gi|386336514	RND transporter	n.p.	n.p.	104	91
gi|307130252	RND efflux membrane fusion protein	n.p.	n.p.	29	42
PA1237	Probable multi-drug resistance efflux pump	9	6	10	0
PA3719	Anti-repressor for MexR, ArmR	20	15	10	33
PA4990	SMR multi-drug efflux transporter	103	79	21	15
PA4374	Probable Resistance-Nodulation-Cell Division (RND) efflux membrane fusion protein precursor	499	444	233	251
PA4375	Probable Resistance-Nodulation-Cell Division (RND) efflux transporter	1024	643	439	293
PA3522	Probable Resistance-Nodulation-Cell Division (RND) efflux transporter	1090	102	381	82
PA3523	Probable Resistance-Nodulation-Cell Division (RND) efflux membrane fusion protein precursor	355	56	140	27
gi|489214414	Outer membrane component of multi-drug efflux pump. partial	0	0	1	1
gi|15596434	Multi-drug resistance efflux pump	0	0	10	0
PA0427	Major intrinsic multiple antibiotic resistance efflux outer membrane protein OprM precursor	2722	2351	991	1137
PA0426	Resistance-Nodulation-Cell Division (RND) multi-drug efflux transporter MexB	7087	5994	2277	3917
PA0425	Resistance-Nodulation-Cell Division (RND) multi-drug efflux membrane fusion protein MexA precursor	3129	2318	1098	1904
PA0424	Multi-drug resistance operon repressor MexR	1305	520	331	321
PA1238	Probable outer membrane component of multi-drug efflux pump	4	7	0	3
PA1435	Probable Resistance-Nodulation-Cell Division (RND) efflux membrane fusion protein precursor	44	40	16	23
PA1436	Probable Resistance-Nodulation-Cell Division (RND) efflux transporter	158	181	70	61
PA0158	Resistance-Nodulation-Cell Division (RND) triclosan efflux transporter, TriC	2826	2341	1241	539
PA0157	Resistance-Nodulation-Cell Division (RND) triclosan efflux membrane fusion protein, TriB	398	433	200	118
PA0156	Resistance-Nodulation-Cell Division (RND) triclosan efflux membrane fusion protein, TriA	530	529	367	177
PA2019	Resistance-Nodulation-Cell Division (RND) multi-drug efflux membrane fusion protein precursor	939	1323	79	70
PA2018	Resistance-Nodulation-Cell Division (RND) multi-drug efflux transporter	3969	4876	321	240
PA2528	Probable Resistance-Nodulation-Cell Division (RND) efflux membrane fusion protein precursor	508	452	349	690
PA2527	Probable Resistance-Nodulation-Cell Division (RND) efflux transporter	603	529	345	572
gi|489252367	Multi-drug transporter	23	35	16	8
PA2526	Probable Resistance-Nodulation-Cell Division (RND) efflux transporter	504	542	594	449
gi|489215922	Resistance-Nodulation-Cell Division (RND) efflux transporter. partial	31	28	n.p.	n.p.
PA2522	Outer membrane protein precursor CzcC	80	17	116	15
PA2521	Resistance-Nodulation-Cell Division (RND) divalent metal cation efflux membrane fusion protein CzcB precursor	184	47	147	20
PA2520	Resistance-Nodulation-Cell Division (RND) divalent metal cation efflux transporter CzcA	369	77	430	51
PA2495	Multi-drug efflux outer membrane protein OprN precursor	91	111	13	6
PA2494	Resistance-Nodulation-Cell Division (RND) multi-drug efflux transporter MexF	1021	767	126	82
PA2493	Resistance-Nodulation-Cell Division (RND) multi-drug efflux membrane fusion protein MexE precursor	110	106	35	61
PA1541	Probable drug efflux transporter	38	104	148	1088
PA5160	Drug efflux transporter	225	208	191	128
PA4206	Probable Resistance-Nodulation-Cell Division (RND) efflux membrane fusion protein precursor	49	54	11	80
PA4207	Probable Resistance-Nodulation-Cell Division (RND) efflux transporter	175	146	58	99
PA4597	Multi-drug efflux outer membrane protein OprJ precursor	42	142	28	197
PA4598	Resistance-Nodulation-Cell Division (RND) multi-drug efflux transporter MexD	153	297	83	964
PA4599	Resistance-Nodulation-Cell Division (RND) multi-drug efflux membrane fusion protein MexC precursor	16	66	31	583
PA3676	Probable Resistance-Nodulation-Cell Division (RND) efflux transporter	385	503	141	233
PA3677	Probable Resistance-Nodulation-Cell Division (RND) efflux membrane fusion protein precursor	24	72	23	70

a n.p. – gene sequence is not present in this strain.

Identifiers state PAO1 gene IDs or RefSeq accession where applicable. Transcriptional activity in tap water (T) and waste water (W) was normalized for the two strains independently and is shown as normalized pseudocounts.

A small set of genes associated with heavy metal tolerance was also found in the genomes of PA30 and PA49 (Fig. 3B; Table 3). However, no differential expression in the two water matrices was detected. Comparing the average expression of these genes with genes not related to metal tolerance also showed no general difference, independent of strain background and water matrix (Fig. 4).

Waste waters are already known to stimulate genetic transfer due to the sublethal noxa of pharmaceutical residues (e.g. antibiotics, heavy metal ions) or other xenobiotics. But, the expression of mobile genetic element was also found to be induced after exposure to tap water. Here, physiological shifts to oligotrophic habitats and/or starvation might be responsible for the genetic activities and might contribute to horizontal gene transfer, as discussed in Davies and colleagues (2006). The genomic analysis identified a large number of mobile genomic elements in the genomes of both PA30 and PA49 (Fig. 3C; Table 4). The genes that can be directly associated with horizontal gene transfer and recombination (recombinases, integrases, transposases and genes related to conjugative transfer) were mostly found to be actively expressed in both strains and independent from the water matrix. On average, these ‘mobility genes’ were expressed on a lower level than the ‘other’ genes of the genome (Fig. 4). However, their expression is insensitive to the strain background and to the water matrix.

In conclusion, the multi-drug resistance of strain PA49 can be attributed to the presence and expression of genes encoding a set of antibiotic-modifying enzymes located both in the core genome and on mobile genetic elements that were presumably acquired by horizontal gene transfer. Thus, the multi-drug resistant phenotype of PA49 seems directly linked with this set of resistance determinants. The impact of one overexpressed efflux pump being induced in waste water on the resistance characteristics of PA49 is so far an open question. Both, the antibiotic resistant and the sensitive strain, showed similar transcriptomic responses to the different water matrices but no strain-specific stress responses to both matrices (with exception to one efflux pump).

## Experimental procedures

### Isolation and cultivation of *P. aeruginosa* strains PA30 and PA49

Bacterial strains were enriched and isolated from a German waste water treatment plant compartment as described in a previous study (Schwartz *et al*., 2006). For routine culturing, bacteria were grown on agar plates containing BM2 minimal medium (Yeung *et al*., 2011) supplemented with 15 g l^−1^ agar (Merck, Darmstadt, Germany). For overnight cultures, a colony from the agar plate was inoculated in BM2 minimal medium as well as BHI (Merck, Darmstadt, Germany) broth (1:4 diluted) and incubated at 37°C. The growth behavior of the strains was observed by diluting overnight cultures to an optical density (OD) of 0.1 in BM2 and BHI medium, incubation at 37°C with gentle agitation for a time span of 24 h and monitoring the OD over time for each strain (Infinite 200 PRO, Tecan, Männedorf, Switzerland). No difference in growth behavior between the two isolates was observed in BM2 and BHI broth respectively (data not shown).

### Antibiotic susceptibility testing PA30 and PA49

Resistance characterization for GM (10 μg disc^−1^), CIP (5 μg disc^−1^), IPM (10 μg disc^−1^), CAZ (30 μg disc^−1^), AN (30 μg disc^−1^), AZ (75 μg disc^−1^) and PT (100/10 μg disc^−1^) was evaluated using agar diffusion test according to Clinical Laboratory Standards Institute (CLSI) guidelines, wherein the zone of growth inhibition on Miller Hinton agar (Merck) was measured after 18 h incubation at 37°C. The *P. aeruginosa* strain PA30 was found to be sensitive for GM, CIP, IPM, CAZ, AN, AZ and PT. In contrast, PA49 was found to be resistant against all mentioned antibiotics (Schwartz *et al*., 2006).

### Incubation in tap water and waste water

Distinct colonies of each strain were inoculated in 25 ml BHI medium (Merck, Darmstadt, Germany) diluted 1:4 with distilled water in a 50 ml sterile tube (Falcon, Nürtingen, Germany) and incubated on a shaker at 37°C at 100 rpm overnight. A volume of 2.5 ml of this overnight culture was used to inoculate 25 ml of 1:4 diluted BHI medium and incubated on a shaker at 37°C at 100 rpm. At an optical density (OD_600nm_) of 1.0 (early stationary growth phase), bacterial suspension were pelleted at 5000 g at 20°C for 15 min. Pellets were re-suspended in 20 ml sterile tap water (T) or sterile filtered waste water (W) collected from the influent of a municipal WWTP. The OD of these suspensions with PA30 and PA49 were adjusted at 0.5. The samples were incubated on a shaker (80 rpm) at 22°C for 3 h.

The tap water conditioned from groundwater at the municipal waterworks met the requirements of the German drinking water guideline. The average total organic carbon value was measured as 0.9 mg l^−1^. The chemical and physical characteristics of the final conditioned drinking water are listed in Jungfer *et al*. (2013; see reference waterworks).

The used waste water originated from the effluent of a municipal waste water treatment plant of a city with 445 000 inhabitants and is equipped with a conventional three treatment process (nitrification, denitrification, phosphor elimination). Chemical analyses demonstrated the presences of different classes of antibiotics (e.g. clarithromycin, roxithromycin, erythromycin, sulfamethoxazol, and trimehoprim) in a range of 0.5–1.5 μg l^−1^ (unpublished data).

### DNA extraction and purification

Previous to the DNA extraction 25 ml BHI was inoculated with a single colony of PA30 and PA49, respectively, and cultivated at 37°C and 150 rpm on a rotary shaker until ODs reached 1.0 value. An aliquot of 5 ml of each culture was pelleted at 3000 g for 10 min. Subsequent DNA extraction was performed according to the protocol of QIAGEN Genomic-tip 100/G kit system (Qiagen, Germany). The concentration and purity of the obtained DNA was determined using the NanoDrop 1000 Spectrophotometer (Thermo Scientific, Germany). The quality of the genomic DNA was also controlled by agarose gel electrophoresis.

### RNA extraction and purification

Ribonucleic isolation of the samples was performed in quadruplicates that were pooled before sequencing. One millilitre of each of the four independent bacterial suspensions (T or W) was mixed with 1 ml of RNA protect (Qiagen, Hilden, Germany) and incubated for 5 min at room temperature. The bacteria were pelleted at 12.000 g for 10 min, and the supernatant was discarded. Prior to RNA extraction from bacteria, four replicate cultures from parallel experiments (tap water and waste water) from each type (PA30 and PA49) were combined. Ribonucleic acid isolation was performed using the RNeasy extraction kit (Qiagen, Hildern, Germany) according to the manufacturer's protocol, and the RNA was eluted in 50 μl RNase-free water. To eliminate residual DNA contamination, the RNA was treated with TURBO Desoxyribonuclease (DNase, Ambion Inc., Kaufungen, Germany). Five microlitres of 10× TURBO DNase buffer and 1 μl of TURBO DNase were added to 50 μl RNA solution and incubated at 37°C for 30 min. Desoxyribonuclease inactivation reagent (5 μl) was added to the RNA solution and incubated under occasional mixing for 5 min. The sample was centrifuged at 10 000 rpm for 1.5 min, and the RNA was transferred to a new tube, and RNA concentration was measured in triplicate using the Nanodrop ND1000 spectrophotometer (PeqLab Biotechnology GmbH, Erlangen, Germany). The integrities of all RNA samples were tested using the Agilent 2100 Bioanalyzer (Agilent Technologies Sales & Services GmbH & Co.KG, Waldbronn, Germany).

### Removal of the ribosomal RNA

Removal of ribosomal RNA (rRNA) was performed with each sample. Fourteen microlitres of total RNA was mixed with 1 μl of RNase inhibitor SUPERase IN (Ambion). Ribosomal RNA was removed with the MICROBExpress KIT (Ambion) according to the manufacturer's protocol. Purified RNA was re-suspended in 25 μl TE buffer (1 mM EDTA, 10 mM Tris, pH: 8.0). The resulting purified mRNA yields were quantified with Nanodrop ND1000.

### Library preparation and Illumina sequencing

Deoxyribonucleic acid sequencing libraries were produced from 1 μg of genomic DNA and RNA libraries from 50 ng of rRNA depleted RNA, following the recommendations of the TruSeq DNA and TruSeq RNA protocols (Illumina) respectively. Briefly, the quality and quantity of ribosomal depleted RNA were assessed with the Bioanalyzer 2100 (Agilent), and the RNA-seq libraries were fragmented chemically, purified with AMPure XP beads (Beckman Coulter) and ligated to adapters with specific DNA barcode for each sample following the Illumina protocol. For DNA-seq libraries, genomic DNA was sheared to 200 bp fragments by sonication with a Covaris S2 instrument using the following settings: peak incidence power 175 W, duty factor 10%, cycle per burst 200, time 430 s. Sizes and concentrations of both RNA and DNA sequencing libraries were determined on a Bioanalyzer 2100 (DNA1000 chips, Agilent). Paired-end sequencing (2 × 50 bp) was performed on two lanes on a Hiseq1000 (Illumina) platform using TruSeq PE Cluster KIT v3 – cBot – HS and TruSeq SBS KIT v3 – HS. Cluster detection and base calling were performed using rtav1.13 and quality of reads assessed with casava v1.8.1 (Illumina). The sequencing resulted in at least 40 million pairs of 50 nt long reads for each sample, with a mean Phred quality score > 35 (Tables S1 and S3). These sequence data have been submitted to the GenBank Sequence Read Archive and are available under the accession numbers SRP041029 (PA30 genome), SRP041030 (PA49 genome), SRP041150 (PA30 transcriptomes) and SRP041151 (PA49 transcriptomes).

### Genome assembly and annotation

Raw sequence reads were trimmed and filtered using the fastq-mcf tool of the ea-utils software package (Aronesty, 2011) with a Phred quality cut-off of 20 and the appropriate Illumina TruSeq adapter sequences to detect and remove adapters. The genomes of strains PA30 and PA49 were assembled independently using the idba_hybrid assembler (Peng *et al*., 2012) with a range of k-mer sizes from 20 to 50 bp (step size 10 bp). The genome reference used to guide the hybrid assembly included the full genomic sequence of *P. aeruginosa* PAO1 and the sequences of 13 genomic islands and one plasmid (Table S2). To confirm the resulting contigs, the filtered and trimmed reads were mapped against the contigs using Bowtie 2 (Langmead and Salzberg, 2012), and the read coverage of each contig was calculated by dividing the number of reads mapping to that contig by its length. Contigs with a length of less than 250 bp or with a read coverage of less than one read per base pair were discarded for being too short and/or potentially artefacts. Thereby, 207 (out of 775) and 269 (out of 887) contigs remained for the genomes of PA30 and PA49 respectively (Table S1). The overlap of the contigs with the reference sequences was determined by multi-sequence alignment using the PROmer function of the MUMmer 3.0 software package (Kurtz *et al*., 2004).

The filtered contigs were scanned for protein coding genes using MetaGeneMark (Trimble *et al*., 2012) with default parameters yielding 6572 and 6781 genes for PA30 and PA49 respectively (Table S1). The genes were annotated in two steps by using a blastp search (Camacho *et al*., 2009) first against the *P. aeruginosa* PAO1 genome with protein sequences taken from the *Pseudomonas* genome database (Winsor *et al*., 2011) and then for the remaining unidentified protein sequences against the database of nr protein sequences available for download from the NCBI FTP site (ftp://ftp.ncbi.nlm.nih.gov/blast/db). Thereby, 65 and 97 of the predicted genes of PA30 and PA49 remained unidentified. Additionally, the predicted protein sequences were compared by blastp to the CARD (McArthur *et al*., 2013) to identify putative resistance genes.

To enable direct comparisons between the genomes of PA30 and PA49, the orthologous genes were determined by reciprocal alignment of the protein sequences using blast. Genes in both genomes that reciprocally yielded the highest blast score for each other in the alignment with at least 80% sequence identity were considered to be orthologs.

### Analysis of gene expression

Raw reads generated from complementary DNA were trimmed and filtered using the fastq-mcf tool of the ea-utils software package with a Phred quality cut-off of 20 and the appropriate Illumina TruSeq adapter sequences to detect and remove adapters. Gene expression was determined by mapping the reads to the newly assembled and contigs using Bowtie 2 and counting the reads that uniquely overlapped with the annotated genes. Differential gene expression was analysed using the r-package DESeq (Anders and Huber, 2010). Gene were considered to be differentially expressed, when the absolute log_2_ fold change was greater than 2 (equivalent to fourfold upregulation or downregulation) with a *P*-value (adjusted for multiple hypothesis testing) below 0.05. A GO enrichment analysis was performed using the Blast2GO software (Conesa *et al*., 2005; Götz *et al*., 2008), testing for the enrichment of GO terms in the set of differentially expressed genes with a false discovery rate (FDR) of less than 0.05.

### Multilocus Sequence Typing

The sequence types of the strains PA30 and PA49 were determined following the multilocus sequence typing (MLST) scheme described by Curran and colleagues (2004), by comparing the sequences of seven variable genes commonly found in *P. aeruginosa* (*acsA*, *aroE*, *guaA*, *mutL*, *nuoD*, *ppsA*, *trpE*) with the public online database PubMLST, http://www.pubmlst.org (Jolley and Maiden, 2010). The sequences of the seven MLST regions were concatenated for both strains and aligned with the concatenated MLST sequences of eight previously published genomes (*P. aeruginosa* strains PAO1, PA14, PA7, PASC2, LESB58, NCGM2, DK2 and M18, all taken from the *Pseudomonas* genome database http://www.pseudomonas.com (Winsor *et al*., 2011) ) using ClustalW (Larkin *et al*., 2007). A phylogenetic tree was constructed from the alignment using ClustalW and TreeView (Page, 1996).

## Conflict of Interest

None declared.

## Supporting Information

**Table S1.** Genome sequencing and assembly statistics. **Table S2.** Coverage of references sequences. Percentages indicate the fraction of the references sequences that overlapped with the newly assembled contigs. See also Fig. 1. **Table S3.** Ribonucleic acid sequencing statistics. **Table S4.** MS Excel table file (.xslx) of genes that were differentially expressed comparing the transcriptomes of isolate PA30 in waste water and tap water. The file contains two table sheets listing the genes that were upregulated or downregulated upon exposure to waste water as compared with exposure to tap water. **Table S5.** MS Excel table file (.xslx) of genes that were differentially expressed comparing the transcriptomes of isolate PA49 in waste water and tap water. The file contains two table sheets listing the genes that were upregulated or downregulated upon exposure to waste water as compared with exposure to tap water.
